# Genetic Basis and Functional Consequences of Differential Expression of the CmeABC Efflux Pump in *Campylobacter jejuni* Isolates

**DOI:** 10.1371/journal.pone.0131534

**Published:** 2015-07-01

**Authors:** Tara Grinnage-Pulley, Qijing Zhang

**Affiliations:** Department of Veterinary Microbiology and Preventive Medicine, College of Veterinary Medicine, Iowa State University, Ames, Iowa, United States of America; Institut National de la Recherche Agronomique, FRANCE

## Abstract

The CmeABC multidrug efflux transporter of *Campylobacter jejuni* plays a key role in antimicrobial resistance and is suppressed by CmeR, a transcriptional regulator of the TetR family. Overexpression of CmeABC has been observed in laboratory-generated mutants, but it is unknown if this phenotype occurs naturally in *C*. *jejuni* isolates and if it has any functional consequences. To answer these questions, expression of *cmeABC* in natural isolates obtained from broiler chickens, turkeys and humans was examined, and the genetic mechanisms and role of *cmeABC* differential expression in antimicrobial resistance was determined. Among the 64 *C*. *jejuni* isolates examined in this study, 43 and 21 were phenotypically identified as overexpression (OEL) and wild-type expression (WEL) levels. Representative mutations of the *cmeABC* promoter and/or CmeR-coding sequence were analyzed using electrophoretic mobility shift assays and transcriptional fusion assays. Reduced CmeR binding to the mutated *cmeABC* promoter sequences or decreased CmeR levels increased *cmeABC* expression. Several examined amino acid substitutions in CmeR did not affect its binding to the *cmeABC* promoter, but a mutation that led to C-terminal truncation of CmeR abolished its DNA-binding activity. Interestingly, some OEL isolates harbored no mutations in known regulatory elements, suggesting that *cmeABC* is also regulated by unidentified mechanisms. Overexpression of *cmeABC* did not affect the susceptibility of *C*. *jejuni* to most tested antimicrobials except for chloramphenicol, but promoted the emergence of ciprofloxacin-resistant mutants under antibiotic selection. These results link CmeABC overexpression in natural *C*. *jejuni* isolates to various mutations and indicate that this phenotypic change promotes the emergence of antibiotic-resistant mutants under selection pressure. Thus, differential expression of CmeABC may facilitate *Campylobacter* adaptation to antibiotic treatments.

## Introduction

Multidrug efflux pumps play key roles in bacterial physiology, conferring intrinsic and acquired resistance to diverse toxic compounds. There are multiple types of drug efflux systems in bacteria, but in gram-negative bacteria, the resistance-nodulation- cell division (RND) family of efflux pumps is of primary importance for antimicrobial resistance [1]. ArcAB-TolC, MexAB-OprM, and MexXY-Z in *Escherichia coli* and *Pseudomonas aeruginosa* are examples of well-characterized RND efflux pumps that extrude bile salts, organic cations, detergents, and various classes of antimicrobials [2–5]. These efflux pumps have been associated with intrinsic and acquired resistance to antimicrobial compounds. CmeABC, also a member of the RND family, is the predominant efflux pump in *Campylobacter jejuni* [6] and plays a key role in the resistance to structurally diverse compounds, such as bile salts, ciprofloxacin, erythromycin, ethidium bromide, and various detergents [6, 7]. Synergistic effects of CmeABC with other resistance mechanisms (such as target gene mutations) contribute to high-level resistance to macrolides, tetracyclines, and fluoroquinolones in *Campylobacter* [8–11]. Due to its significant role in bile resistance, CmeABC is essential for *Campylobacter* colonization and adaptation in the intestinal tract of animals [7].

CmeABC is comprised of an inner membrane transporter (CmeB), a periplasmic fusion protein (CmeA), and an outer membrane protein (CmeC). The three components are encoded by a three-gene operon [6]. Transcription of this operon is repressed by CmeR, a TetR family transcriptional regulator [12]. The *cmeR* gene is located immediately upstream of the *cmeABC* operon. The CmeR protein contains a C-terminal ligand-binding domain and a N-terminal DNA-binding domain [7]. The DNA-binding domain interacts specifically with a 16-base inverted repeat within the promoter region of the *cmeABC* operon [13]. This binding inhibits the transcription of the *cmeABC* operon. However, mutation of CmeR or alteration of the promoter sequences affects the binding of CmeR, resulting in increased expression of CmeABC [12, 14]. Additionally, *cmeABC* expression is inducible by bile and this induction is through the interaction of bile with the ligand-binding pocket of CmeR, which triggers a conformational change in the DNA-binding domain, releasing CmeR from the *cmeABC* promoter [15, 16].

Gastroenteritis caused by *Campylobacter* is estimated to affect 845,024 people and cause 8,463 hospitalizations per year in the United States [17]. *C*. *jejuni* and *C*. *coli* are the most common *Campylobacter* species associated with foodborne disease and are commensals in avian species (particularly poultry), swine, and ruminants [18]. *Campylobacter* contamination frequently occurs with poultry meat and unprocessed milk. Thus, undercooked poultry and unpasteurized milk are common vehicles for foodborne transmission of *Campylobacter* to humans [18, 19]. Clinically, campylobacteriosis is manifested as diarrhea, abdominal cramps, and fever, which typically resolves in 1 week without medical intervention. However, when antimicrobial treatment is indicated with severe or prolonged cases, or in immunocompromised patients, fluoroquinolones and macrolides are the drugs of choice [18, 20]. Increasing resistance to these antibiotics in *Campylobacter* is problematic, especially to fluoroquinolones, as *Campylobacter* is highly adaptable to fluoroquinolone treatment and acquisition of mutations associated with fluoroquinolone resistance does not impose a fitness cost on this organism [21–23]. In all clinically relevant antibiotic resistance, CmeABC plays an important role as inactivation of *cmeABC* rendered *Campylobacter* much more susceptible to various antimicrobials [6].

Considering the significance of CmeABC in *Campylobacter* pathobiology, its varied expression levels are expected to affect antimicrobial resistance. Under toxic conditions or in the adaptation to harsh environments, enhanced expression of *cmeABC* may confer a survival advantage on *Campylobacter*. The advantage may occur directly through increased extrusion of toxic substrates, which reduces their harmful effect and increased frequency of emergence of antimicrobial resistant mutants. Although inactivation of *cmeABC* or overexpression of this efflux pump has been examined under experimental conditions by using insertional mutagenesis or stepwise selection of mutants on antibiotic containing plates [12, 14], it is unknown if differential expression of *cmeABC* occurs in naturally occurring isolates and if the differential expression has any functional consequences. In this study, we investigated the expression of *cmeABC* in *C*. *jejuni* isolates from turkeys, chickens, and humans, examined the mechanisms associated with the differential expression, and measured the functional consequences associated with the differential expression.

## Materials and Methods

### Bacterial strains and growth conditions

Sixty-four naturally occurring *Campylobacter* isolates were originally derived from conventionally raised broiler chickens [24], conventionally raised turkeys [24], and clinical diarrheal cases of humans [25]. These isolates were retrospective collections used in previous publications [24, 25]. The 20 broiler isolates, 17 turkey isolates, and 27 human isolates were confirmed to be *C*. *jejuni* using the reported *mapA* and 16S rRNA primers [26, 27]. Key PCR primers used in this study are listed in Table 1. In addition to these isolates, several laboratory strains were also used, including NCTC 11168 [28], 81–176 [29], 81–176Δ*cmeR* [12], 11168Δ*cmeR* [12] and the quality control *C*. *jejuni* strain ATCC 33560 [30, 31], which are listed in Table 2. All strains were routinely cultured in Mueller Hinton (MH) agar or MH broth (Difco, Detroit, MI) at 42°C under microaerobic conditions (5%O_2_, 10%CO_2_, 85%N_2_). Media were supplemented with kanamycin at 30 μg/mL or chloramphenicol at 4 μg/mL when needed.

**Table 1:** Oligonucleotide primers used for PCR and real time RT-PCR.^1^

Primer Type	Primer Name	Sequence	Source or Reference
PCR			
	RIGA2-F	CAAGTTTAGCAGGGTAAGTAA	This study
	RIGA2-R	TAAATTAAAAGCAGGAGAACAAG	This study
	16srRNA-F	AATCTAATGGCTTAACCATTA	[26]
	16srRNA-R	GTAACTAGTTTAGTATTCCGG	[26]
	*mapA*-F	GAGTGCTTGTGCAACTAAAC	[27]
	*mapA*-R	ATAGCATCTTGAGTTGCTCC	[27]
	GSF	CTAAATGGAATCAATAGCTCC	[12]
	GSR1	GCACAACACCTAAAGCTAAAA	[12]
	PF	AAAAGGATCCTAAATGGAATCAATAGCTCC (*BamHI)*	[12]
	PX	GCGGCATTTGTATTTCTAGAGCTTCTTCT (*XbaI)*	This study
	pMW10-F	ATCTGCCTCCTCATCCTCTTCAT	This study
	pMW10-R	ATTCAGGCTGCGCAACTGTT	This study
	M63R17-F	ATGAACTCAAATAGAATACCATCACAAAAAGTT	This study
	M63R17-R	AACTTTTTGTGATGGTATTCTATTTGAGTTCAT	This study
	M63R475-F	TATATGAAAAAAAATGCAAAAAAACTTGCTGTTCTTT	This study
	M63R456-R	AAAGAACAGCAAGTTTTTTTGCATTTTTTTTCATATA	This study
	T3X250A-F	CCAAAACACAAGAAATTAAAAATGGCACTTTAAAA	This study
	T3X250A-R	TTTTAAAGTGCCATTTTTAATTTCTTGTGTTTTGG	This study
	T97547G-F	AATGTTTTAATTAACGCTGCTTTGAAAAATAAAAAAG	This study
	T97547G-R	CTTTTTTATTTTTCAAAGCAGCGTTAATTAAAACATT	This study
	T22583-F1	GAACATGTTTGAATTTGTTGTAAATGTTTTT	This study
	T22583-R1	AAAAACATTTACAACAAATTCAAACATGTTC	This study
	r431GA-F	CTATAACATACTTATGGATTTTTTCAAGCAACAAA	This study
	r431GA-R	TTTGTTGCTTGAAAAAATCCATAAGTATGTTATAG	This study
	r619621-F	AATGGAATCAATGGATCCAAAGCTTAA	This study
	r619621-R	TTAAGCTTTGGATCCATTGATTCCATT	This study
	pQETypeIII/I-F	CG GATAACAATT TCACACA G	Promega
	pQEReverse-R	GTTCTGAGGTCATTACTGG	Promega
	Cj0370-F1	CAGTCCTCACCACCTTTCT	This study
	Cj0368c-R	AGGCCACTGCTTTGATT	This study
	370BamH-F	CAGTCGGATCCACCTTTC (*BamHI)*	This study
	369XbaI-R1	AAATATCGTTTTTTTCTAGAGTTTGTAAT (*XbaI)*	This study
Real time RT-PCR			
	16S-F	TACCTGGGCTTGATATCCTA	[34]
	16S-R	GGACTTAACCCAACATCTCA	[34]
	*cmeB*-F	ACGATTCAACCTTTTCCCAGC	[34]
	*cmeB*-R	TTTGCTACTTGAGCAATCGCTTC	[34]
	F3	ATTTTCAATCAACCAGAAGCTG	[16]
	R3	TCCAATTGGCAAGATGTCTATC	[16]

1 Restriction sites are indicated by underlined sequence

**Table 2:** Bacterial strains or plasmids used in this study.

Plasmid or Bacteria	Plasmid or Strain Name	Description	Source
Plasmids			
	pMW10	*E*. *coli–Campylobacter* shuttle vector carrying promoter-less *lacZ*, KanR	[32]
	pMW11168	pMW10 carrying the *cmeABC* promoter from NCTC11168 fused to *lacZ*, KanR	This study
	pMW81-176	pMW10 carrying the *cmeABC* promoter from 81–176 fused to *lacZ*, KanR	This study
	pMWX7199	pMW10 carrying the *cmeABC* promoter from X7199 fused to *lacZ*, KanR	This study
	pMWM32506	pMW10 carrying the *cmeABC* promoter from isolate M32506 fused to *lacZ*, KanR	This study
	pMW1:1	pMW10 carrying the *cmeABC* promoter from isolate CT1:1 fused to *lacZ*, KanR	This study
	pMW1:9	pMW10 carrying the *cmeABC* promoter from isolate CT1:9 fused to *lacZ*, KanR	This study
	pMW3:7	pMW10 carrying the *cmeABC* promoter from isolate CT3:7 fused to *lacZ*, KanR	This study
	pMW9:20	pMW10 carrying the *cmeABC* promoter from isolate CT9:20 fused to *lacZ*, KanR	This study
	pMW11168-R	pMW10 carrying the *Cj0369c-cmeR* promoter from NCTC 11168 fused to *lacZ*, KanR	This study
	pMW81-176-R	pMW10 carrying the *Cj0369c-cmeR* promoter from 81–176 fused to *lacZ*, KanR	This study
	pMWX7199-R	pMW10 carrying the *Cj0369c-cmeR* promoter from isolate X7199 fused to *lacZ*, KanR	This study
	pQE30	Expression vector for N-terminal 6-His tagged proteins, AmpR	Qiagen
	pQECmeRSS	pQE30 carrying CmeR with the C69S and C166S mutations	[37]
	pQECmeR-K	pQE30 carrying CmeRSS with the E84K mutation, AmpR	This study
	pQECmeR-R	pQE30 carrying CmeRSS with the P183R mutation, AmpR	This study
	pQECmeR-IK	pQE30 carrying CmeRSS with the T6I and E159K mutations, AmpR	This study
	pQECmeR-tr	pQE30 carrying CmeRSS with the G144A and S207G amino acid mutations. Also carries T insertion at nucleotide 583 causing frame shift after amino acid 193, AmpR	This study
*Campylobacter jejuni* strains			
	NCTC 11168	Wild type; genome sequence known	[1]
	11168Δ*cmeR*	Derivative of NCTC 11168, *cmeR*::*cat*	[2]
	ATCC 33560	C. *jejuni* quality control strain	[3, 4]
	81–176	Wild type; isolated from a human	[5]
	81-176pMW10	Derivative of 81–176 carrying pMW10	This study
	81-176pMW11168	Derivative of 81–176 carrying pMW11168	This study
	81-176pMW81-176	Derivative of 81–176 carrying pMW81-176	This study
	81-176pMWX7199	Derivative of 81–176 carrying pMWX7199	This study
	81-176pMWM32506	Derivative of 81–176 carrying pMWM32506	This study
	81–176 pMW1:1	Derivative of 81–176 carrying pMW1:1	This study
	81–176 pMW1:9	Derivative of 81–176 carrying pMW1:9	This study
	81–176 pMW3:7	Derivative of 81–176 carrying pMW3:7	This study
	81–176 pMW9:20	Derivative of 81–176 carrying pMW9:20	This study
	81–176Δ*cmeR*	Derivative of 81–176, *cmeR*::*cat*	[2]
	81–176Δ*cmeR* pMW10	Derivative of 81–176Δ*cmeR* carrying pMW10	This study
	81–176Δ*cmeR* pMW11168	Derivative of 81–176Δ*cmeR* carrying pMW11168	This study
	81–176Δ*cmeR* pMW81-176	Derivative of 81–176, *cmeR*::*cat* carrying pMW81-176	This study
	81–176Δ*cmeR* pMWX7199	Derivative of 81–176Δ*cmeR* carrying pMWX7199	This study
	81–176Δ*cmeR* pMWM32506	Derivative of 81–176Δ*cmeR* carrying pMWM32506	This study
	81–176Δ*cmeR* pMW1:1	Derivative of 81–176Δ*cmeR* carrying pMW1:1	This study
	81–176Δ*cmeR* pMW1:9	Derivative of 81–176Δ*cmeR* carrying pMW1:9	This study
	81–176Δ*cmeR* pMW3:7	Derivative of 81–176Δ*cmeR* carrying pMW3:7	This study
	81–176Δ*cmeR* pMW9:20	Derivative of 81–176Δ*cmeR* carrying pMW9:20	This study
	81-176pMW11168-R	Derivative of 81–176 carrying pMW11168-R	This study
	81-176pMW81-176-R	Derivative of 81–176 carrying pMW81-176-R	This study
	81-176pMWX7199-R	Derivative of 81–176 carrying pMWX7199-R	This study
*Escherichia coli* strains			
	DH5α	F-Φ80lacZΔM15 Δ(lacZYA-argF) U169 recA1 endA1 hsdR17 (tκ,-mκ+) phoA supE44λ- thi-1 gyrA96 relA1	Invitrogen
	DH5αpRK2013	IncP KmR Tra RK2+ Δ*rep*RK2 *rep*E1+	[6]
	DH5αpMW10	DH5α derivative carrying pMW10	[7]
	JM109	e14-(McrA-) recA1 endA1 gyrA96 thi-1 hsdR17(tκ-mκ+) supE44 relA1 Δ(lac-proAB) [F’ traD36 proAB lacqZΔM15]	Agilent
	XL-1 Blue	recA1 endA1 gyrA96 thi-1 hsdR17 supE44 relA1 lac [F’ proAB lacIqZΔM15 Tn10 (Tetr)	Agilent
	JM109pQECmeRSS	Derivative of JM109 carrying pQECmeRSS AmpR	[1]
	JM109pQECmeR-R	Derivative of JM109 carrying pQECmeR-R, AmpR	This study
	JM109pQECmeR-IK	Derivative of JM109 carrying pQECmeR-IK, AmpR	This study
	XL1-Blue pQECmeR-tr	Derivative of XL1-Blue carrying pQECmeR-tr, AmpR	This study

*E*. *coli* strains (Table 2) DH5α (Invitrogen), DH5αpMW10 [32], DH5αpRK2013 [33], XL1-Blue (Agilent), and JM109 (Agilent) were cultured at 37°C. Luria-Bertani (LB) broth or agar (Difco) was supplemented with 30 μg/mL of kanamycin or 100 μg/mL of ampicillin when needed.

### Immunoblotting

Isolates were initially screened for CmeABC expression by immunoblotting with polyclonal antibodies against CmeABC. All clinical *C*. *jejuni* isolates, NCTC 11168 and 11168Δ*cmeR* were cultured in MH broth. Samples were pelleted, and re-suspended in SDS loading buffer for a final concentration of 5 x 10^9^ CFU/mL. The protein samples were analyzed by SDS-PAGE and immunoblotting as described previously using antibodies against CmeA, CmeB, CmeC [6] and MOMP [9]. Bands for CmeA, CmeB, and CmeC from the clinical isolates were compared to those of NCTC 11168 and 11168Δ*cmeR* by visual inspection and densitometric analysis using the AlphaEaseFC Software (Version 3.2.3 Rev C; Innotech). Primary classification as wild type-level (WEL) or overexpression level (OEL) of CmeABC was based on analysis of CmeB by densitometry and CmeA was utilized as a secondary factor for the classification. The 64 clinical *C*. *jejuni* isolates were analyzed on 8 immunoblots with NCTC 11168 and 11168Δ*cmeR* used as controls for WEL and OEL, respectively. The threshold for CmeABC overexpression based on densitometric analysis was a 2-fold increase for the CmeB band in relation to the expression level in NCTC 11168. Each immunoblot was examined individually to ensure the threshold for overexpression was met. This phenotypic classification was further confirmed by measuring the *cmeB* transcript using real time RT- PCR. OEL isolates showed overexpression of CmeB on immunoblotting and/or real time RT-PCR expression of *cmeB*. CmeA expression based on a threshold of 2-fold for overexpression was used for isolates that remained between the two phenotypic groups after real time *cmeB* expression levels were analyzed.

The expression level of CmeR was also evaluated by immunoblotting in selected clinical isolates that harbored mutations in the *cmeR* gene. The whole cell samples were prepared from isolates M63885, CT9:7, CB2:6, CB2:8, CB2:11, S13530, T37957A, X7199, CT2:2, NCTC 11168, and 11168Δ*cmeR*. Samples were loaded onto a 12% SDS PAGE gel for electrophoresis in Lameilli buffer at 80V for 30 minutes followed by 200V for 60 minutes. The gel and PVDF membrane were equilibrated in Towbin transfer buffer for 30 minutes, assembled onto the transfer apparatus, and transferred at 60 V for 40 minutes in Towbin transfer buffer. The membrane was blocked in blocking buffer (5% skim milk with 0.01% Tween-20 in PBS) at 4°C on a rocker. Then it was incubated with rabbit anti-CmeR diluted 1:100 in blocking buffer for 90 minutes at room temperature on a rocker. The membrane was washed 3 times for 10 minutes in washing buffer (0.01% Tween-20 in PBS) and further incubated with goat anti-rabbit IgG conjugated with horseradish peroxidase (KPL) (1:1000 in blocking buffer) for 1 hour at room temperature. After three washings, the membrane was developed with the 4CN Horseradish Peroxidase Substrate system (KPL). Densitometric analysis for CmeR was performed in the same manner for CmeABC.

SDS-PAGE and immunoblotting were also performed for the recombinant mutant CmeR named rCmeR-tr to determine if the protein was recognizable by CmeR antibodies. This recombinant protein was derived from mutations in strain CT2:2 (Table 3). The recombinant CmeR named rCmeRSS was used as a control. Both rCmeRSS and rCmeR-tr were loaded at 400 ng onto a 12% SDS-PAGE gel. Electrophoresis and immunoblotting were performed using the same methods used for immunoblotting of CmeR in clinical isolates.

**Table 3:** Mutation, expression, and phenotypes of CmeR in selected *C*. *jejuni* isolates.

Clinical Isolate	*cmeR* Expression Fold Change*	Nucleotide Mutation	Amino Acid Mutation	Recombinant Protein	Binding to *cmeABC* promoter
CT2:2	0.006	G431A	G144A	rCmeR-tr	No
		583 T insertion	Truncation after 193		
		A619G	S207G		
		C621A	Silent		
M63885	0.845	C17T	T6I	rCmeR-IK	Yes
		G475A	E159K		
T37957A	33.107	G250A	E84K	rCmeR-K	Yes
CT9:7	0.378	C547G	P183R	rCmeR-R	Yes
CB2:8	1.117	G431A	G144A	Not tested	Not tested
CB2:11	0.327	C547G	P183R		
CB2:6**	0.806	A619G	S207G		
S13530	0.184	C621A	silent		
X7199	10.861	None	None	Not tested	Not tested

*In relative to the expression level in NCTC 11168 as determined by RT-PCR

**Isolate is phenotypically classified as WEL

### Real time RT-PCR

Transcription of *cmeB* and *cmeR* was detected by real time RT-PCR to confirm the results of immunoblotting. RNA was isolated from 24-hour cultures of clinical isolates, NCTC 11168, and 11168Δ*cmeR* as described previously (13). Real time RT-PCR was performed for all isolates for *cmeB* and for selected isolates for *cmeR* as described previously (12, 13, 16, 34). The primers used for real time RT-PCR are shown in Table 1. Relative expression based on NCTC 11168 was calculated with the Pfaffl Method [35]. Overexpression of *cmeB* was defined as greater or equal to 3 fold of NCTC 11168 expression. *cmeB* expression measured by RT-PCR was compared with the result of immunoblotting for final determination of a CmeABC phenotype. For those isolates where RT-PCR and immunoblotting data did not align, less weight was given to real time RT-PCR data due to reported variability in *cmeB* coding sequence [36].

### DNA sequencing

DNA sequencing was performed to determine if isolates were carrying mutations in *cmeR*, the *Cj0369c-cmeR* promoter or the *cmeABC* promoter. All clinical isolates classified as having a phenotype of CmeABC overexpression, some selected clinical isolates with the wild-type expression levels, and 81–176 were amplified with RIGA-F and RIGA-R primers (Table 1) for sequence analysis of the region between *cmeR* to the 5’end of *cmeA*, which covers the whole ORF of *cmeR* and the entire promoter of *cmeABC*. Additionally, the predicted promoter region of *Cj0369c-cmeR* (the two genes share a single promoter located upstream of *Cj0369c*) was examined to determine if mutations in the promoter region were involved in differential CmeR expression in clinical isolates. The *Cj0369c-cmeR* promoter was amplified with *Cj0370*-F and *Cj0368c*-R (Table 1). GenBank accession numbers are found in S1 Table.

### Electrophoretic mobility shift assays (EMSA)

EMSA was used to assess the binding of CmeR to the *cmeABC* promoter sequences from various clinical isolates. These clinical isolates were observed to contain sequence polymorphisms within the *cmeABC* promoter region. A recombinant CmeR named rCmeRSS [37] with cysteines 69 and 166 replaced with serine was used for EMSA as described previously [12, 38]. The Cys-Ser substitution does not affect the binding activity, but significantly improves the stability of recombinant CmeR as the Cys residues are sensitive to oxidation during *in vitro* binding assay. The 170-bp *cmeABC* promoter sequences were amplified from genomic DNA of NCTC 11168, 81–176, CT3:7, CT1:1, CT1:9, CT9:20, and X7199 with primers GSF and GSR1[12] (Table 1). The amplified products were purified (QIAquick PCR Purification kit, Qiagen) and then labeled with DIG-11-dd-dUTP using the DIG Oligonucleotide 3’ End Labeling kit (Roche). The labeled promoter DNA was used as probes in EMSA.

The *cmeABC* promoter probes were named for their strain of origin: 11168, 81–176, CT3:7, CT1:1, CT1:9, CT9:20 and X7199. Promoter probes from clinical isolates (CT3:7, CT1:1, CT1:9, CT9:20 and X7199) were compared with the promoter probes of 11168 or 81–176 (laboratory strains), depending on their similarity to the CmeR binding site in the two laboratory strains. The CmeR-binding site contains an A to T substitution in the 81–176 strain compared to 11168, which is considered a naturally occurring variation [12].

The promoter probes (0.05 pmol each) were incubated with 0, 60, 120, and 180 ng of rCmeRSS in 22 μL of binding buffer according to the method of Alekshun *et*. *al*. and Lin *et*. *al*. 2005 [12, 38]. The reaction mixtures were incubated for 30 minutes at room temperature and Promega DNA loading buffer was added to each reaction. Samples were separated by electrophoresis at 200V for 45 minutes on a 6% polyacrylamide gel in 0.25X TBE Buffer and transferred to a positively charged nylon membrane by vacuum [12]. Chemiluminescent detection using alkaline phosphatase-conjugated anti-DIG antibody and CDP-Star (Roche) was performed as previously described [12].

### Construction of promoter fusions and β-galactosidase assays

The observed sequence polymorphisms in the promoter sequences of *cmeABC* were assessed for their impact on *cmeABC* transcription by constructing transcriptional fusions with a promoter-less *lacZ* gene. Genomic DNA templates (NCTC 11168, 81–176, CT1:1, CT 1:9, CT9:20, CT3:7, M32506 and X7199) were used for amplification of a 578-bp sequence containing the *cmeABC* promoter with primers PF [12] and PX (Table 1). These PCR products were purified (QIAquick PCR Purification kit), digested with *XbaI* and *BamHI* (Promega), and re-purified using the QIAquick kit. Vector pMW10 [32] was purified (QIAprep Spin Miniprep kit, Qiagen) from DH5αpMW10 (Table 2), digested with the same enzymes, and re-purified. Vector and PCR product inserts were ligated with T4 ligase (Roche) and transformed into DH5α. Transformants were selected on LB agar plates supplemented with kanamycin (30 μg/mL). Plasmid constructs (Table 2) were purified from DH5α and sequenced with pMW10-F and pMW10-R (Table 1) to confirm the appropriate sequences and fusion.

To transfer the plasmids into *C*. *jejuni*, tri-parental mating using *C*. *jejuni* 81–176, *E*. *coli* DH5αpRK2013 [33], and the various DH5α pMW10 transcriptional fusion constructs was performed as described previously [33]. After transfer into *C*. *jejuni* 81–176, plasmids pMW11168, pMW81-176, pMW1:1, pMW1:9, pMW3:7, pMW9:20, pMWM32506, and pMWX7199 (Table 2) were purified and electroporated into 81–176Δ*cmeR*. The empty vector, pMW10, was also transferred to 81–176 and 81–176Δ*cmeR* by the same methods and used as a background control. Cultures were grown for 20 hours in MH broth supplemented with kanamycin (30 μg/mL), then β-galactosidase assays were performed as described previously [39]. Three independent experiments were conducted. Student’s t-test with Welch’s correction was used to compare the expression data from various promoters and was done using GraphPad InStat (Version 3.06) with the significance level set at 0.05.

Sequence polymorphisms were also observed in the *Cj0369c-cmeR* promoter. To determine if these mutations affected CmeR expression, a second set of promoter fusions was created using the same method. Briefly, primers 370BamH-F and 369XbaI-R1 (Table 1) were used to amplify a 238-base pair segment containing the *Cj0369c-cmeR* promoter from genomic DNA templates of NCTC 11168, 81–176 and X7199. Construction of plasmids, transformation into DH5α and sequencing was performed as described above. Plasmid constructs pMW11168-R, pMW81-176-R and pMWX7199-R were electroporated into *C*. *jejuni* 81–176 (Table 2). The empty vector, 81-176pMW10, from the prior assay was used as a background control. β-galactosidase assays for 3 independent experiments and statistical analysis were performed as described in the assays with the *cmeABC* promoters.

### Construction, purification, and functional analysis of various CmeR variants

Sequence polymorphisms were detected in CmeR among the analyzed isolates. To determine if the sequence variations affected the DNA binding activity of CmeR, we generated various forms of recombinant CmeR using site-directed mutagenesis. Plasmid pQECmeRSS [37] was used as a template for site directed mutagenesis, which was done using the Stratagene QuikChange II kit. Site-specific primers (Table 1) were used to produce the amino acid changes in CmeR from isolates CT2:2, M63885, CT9:7, and T37597A. All amino acid substitutions, corresponding nucleotide sequences and protein names are listed in Table 3. Isolate M63885 contains 2 amino acid substitutions in CmeR, which were introduced simultaneously into the pQECmeRSS template. The M63R17-F and M63R17-R primers were used to mutate the threonine to isoleucine at residue 3 and the M63475-F and M63475-R primers were used to mutate the glutamate to lysine at residue 159. This mutated plasmid was named pQECmeR-IK. Isolates CT9:7 and T37957A both contain a single amino acid substitution in CmeR. Primers T97547G-F and T97547G-R were used to change the proline to arginine at residue 159 as observed in strain CT9:7, creating the plasmid named pQECmeR-R. Primers T3X250A-F and T3X250A-R introduced the glutamate to lysine substitution at residue 84 as observed in strain T37957A. This plasmid was named pQECmeR-K. Isolate CT2:2 contains 2 amino acid substitutions in CmeR and a nucleotide insertion. This mutated CmeR was created using 2 rounds of mutagenesis. The T insertion after nucleotide 583 was introduced into template pQECmeRSS with primers T22583-F1 and T22583-R1 along with the first amino acid substitution at residue 144, a glycine to alanine substitution, with primers r431GA-F and r431GA-R to create an intermediate plasmid. The mutations were confirmed in the intermediate plasmid prior to introduction of the final mutations. In the second round of mutagenesis, this intermediate was used as a template to introduce the final amino acid change, a serine to glycine substitution at residue 207 with primers r619621-F and r619621-R to create plasmid pQECmeR-tr.

All mutations were introduced into the respective templates by one cycle of 95°C for 30 seconds followed by 16 cycles of 95°C for 30 seconds, 55°C for 30 seconds, and 68°C for 4 minutes. The amplified product was cooled on ice for 2 minutes before *Dpn*-I digestion of parental DNA at 37°C for 1 hour. Each product was transformed into JM109 or XL1-Blue (Agilent) and the transformants were selected on LB agar supplemented with ampicillin (100 μg/mL). The specific mutations were confirmed by sequencing with primers pQETypeIII/IV-F and pQEReverse-R (Table 1).

The CmeR variants including rCmeR-tr, rCmeR-R, rCmeR-K, and rCmeR-IK were induced and purified from their respective *E*. *coli* strains (Table 2) under native conditions [40]. After purification, proteins were desalted using PD-10 desalting columns (GE Healthcare). Proteins of rCmeR-tr and rCmeR-K were concentrated in PBS using Amicon Centricon YM-10 Columns (Millipore).

For functional analysis, EMSA was used to assess the ability of the mutated versions of CmeR to bind to the *cmeABC* promoter. Binding by the purified mutant proteins rCmeR-tr, rCmeR-R, rCmeR-K or rCmeR-IK was compared to binding by the rCmeRSS protein. The 11168 *cmeABC* promoter probe (0.05 pmol) was incubated with 0, 60, 120, and 180 ng of rCmeRSS or one of the mutant rCmeR proteins in 22μL of reaction buffer according to the method of Alekshun *et*. *al*. and Lin *et*. *al*. 2005 [12, 38]. The reaction mixtures were incubated for 30 minutes at room temperature and Promega DNA loading buffer was added to each reaction. Electrophoresis, transfer, and detection were performed using the same methods as described for the EMSA assay.

### Antimicrobial susceptibility testing

Agar dilution test was performed in MH agar and MH agar supplemented with 12,500 μg/mL of sodium choleate according to the CLSI-recommended method [31]. Ciprofloxacin, kanamycin, erythromycin, tetracycline, ampicillin, clindamycin, chloramphenicol, ethidium bromide, sodium choleate, cholic acid and taurocholic acid were tested. In addition to the 21 WEL and 43 OEL clinical isolates, 3 laboratory strains classified as WEL isolates, NCTC 11168, ATCC33560, and 81–176, were also tested. At least 2 experiments were performed for each isolate. Distribution of the minimum inhibitory concentration (MIC) around the median was tested with the Brown Forsythe test (SAS version 9.2) comparing the 43 OEL and 24 WEL isolates. The significance level set at 0.05.

### Fluctuation assays

Fluctuation assays were performed to assess if CmeABC expression levels affected the spontaneous mutation rate to ciprofloxacin in *C*. *jejuni*. The assays were conducted using the methods described by Luria and Delbrück [41] with some modifications [42–46]. Selected WEL isolates (NCTC 11168, 81–176, CB8:14, CT10:18, H2958, CB6:8) and OEL isolates (CT9:7, CB4:22, M76297, CT9:14, CB3:1, T37957A, 11168Δ*cmeR*, 81–176Δ*cmeR*) were cultured on antimicrobial-free MH plates, adjusted to 10^8^ CFU/mL in MH broth, and serially diluted to 10^4^. Thirty-six parallel cultures of 200 μL were inoculated on a 96-well plate and incubated for 24 hours. Total cell counts were determined by plating on antimicrobial free MH agar while spontaneous mutant counts were plated on MH agar supplemented with 4 μg/mL of ciprofloxacin.

Total cell counts for each set of cultures (36 parallel cultures) were estimated from 5 random wells. The 5 wells were selected using the Random Integer Set Generator (Random.org). From each of these wells, a 10 μL sample of culture was removed and serially diluted to 10^−7^. A 90 μL sample from the 10^−5^, 10^−6^, and 10^−7^ dilutions was spread to MH agar. Plates were incubated for 2 days for total CFU counts. Counts from the 5 random wells were averaged to determine the total count for each set of 36 parallel cultures.

To determine the number of spontaneous mutants, all 36 wells were plated to MH agar containing 4 μg/mL of ciprofloxacin. Plates were checked after 2 days for colony size and incubated for an additional day to ensure colonies were large enough for counting. Mutation rate was calculated by the Ma-Sandri-Sarkar Maximum Likelihood Estimator method using the Fluctuation Analysis Calculator [47]. Mutations per culture (m) for WEL and OEL were transformed by the natural logarithm (ln(m)) and compared with the Student’s t-test using GraphPad InStat (Version 3.06) with the significance level set at 0.05.

### *In vitro* ciprofloxacin treatment

Inactivation of CmeABC reduced the emergence of ciprofloxacin-resistant mutants in *C*. *jejuni* under antibiotic selection, while inactivation of *cmeR* (resulting in overexpression of *cmeABC*) increased mutant emergence, suggesting that expression levels of CmeABC influences the emergence of antibiotic resistant mutants. To assess if differential expression of *cmeABC* in naturally occurring isolates affects their adaptation to antibiotic treatment, we examined the emergence frequencies of ciprofloxacin-resistant mutants in isolates of different CmeABC phenotypes. Three WEL (CB6:8, F15871, CT10:18) and three OEL isolates (T37957A, CT7:20, CB8:14) were used for this experiment, which were cultured on non-selective media (MH agar) and then treated with 4 μg/mL of ciprofloxacin using the method of Han *et*. *al*. 2008 [48] with some modifications. Briefly, MH broth supplemented with 4 μg/mL of ciprofloxacin was inoculated to an initial concentration of 10^7^ CFU/mL in a 20 mL of culture with 3 replicates per isolate. Cultures were incubated for 3 days. Samples (0.5 mL) were taken on days 0, 1, 2, and 3—post inoculation for enumeration of ciprofloxacin-resistant mutant and total cell counts. Total counts were cultured on MH agar according to the plate drop method [49] for days 0, 1, 2, and 3. For days 0 to 2 mutants were counted by direct plating 100 μL of culture onto MH-ciprofloxacin (4 μg/mL) agar in duplicate and according to the plate drop method [49] onto MH-ciprofloxacin (4 μg/mL) agar. For day 3, total plate counts and mutants were all determined by the plate drop method. The serial dilutions used in the plate drop method were dilutions 10^−2^ to 10^−7^ for day 0 and 10^−3^ to 10^−8^ for days 1 to 3. Two independent experiments were performed for each isolate. The cell counts were calculated for each culture and log transformed. Data analysis was performed with Graph Pad Prism (Version 6.0c) with multiple unpaired t-tests and Holm-Šídák method for multiple comparisons. The significance level was set at 0.05.

In the second experiment, cultures were inoculated to an initial density of 10^6^ CFU/mL. Samples were collected in the same manner as the first experiment. For each day total counts were cultured on MH agar according to the plate drop method [49]. Mutants were counted by direct of plating 100 μL of culture to MH-ciprofloxacin (4 μg/mL) agar in duplicate and serial dilutions for plate drop method [49] onto MH-ciprofloxacin (4 μg/mL) agar. The serial dilutions used in the plate drop method were dilutions 10^−2^ to 10^−7^ for day 0 and 10^−3^ to 10^−8^ for days 1 to 3. Two independent experiments were performed for each isolate. Data analysis was performed as described in the prior experiment.

## Results

### Phenotypic classification of isolates

**Fig 1:**
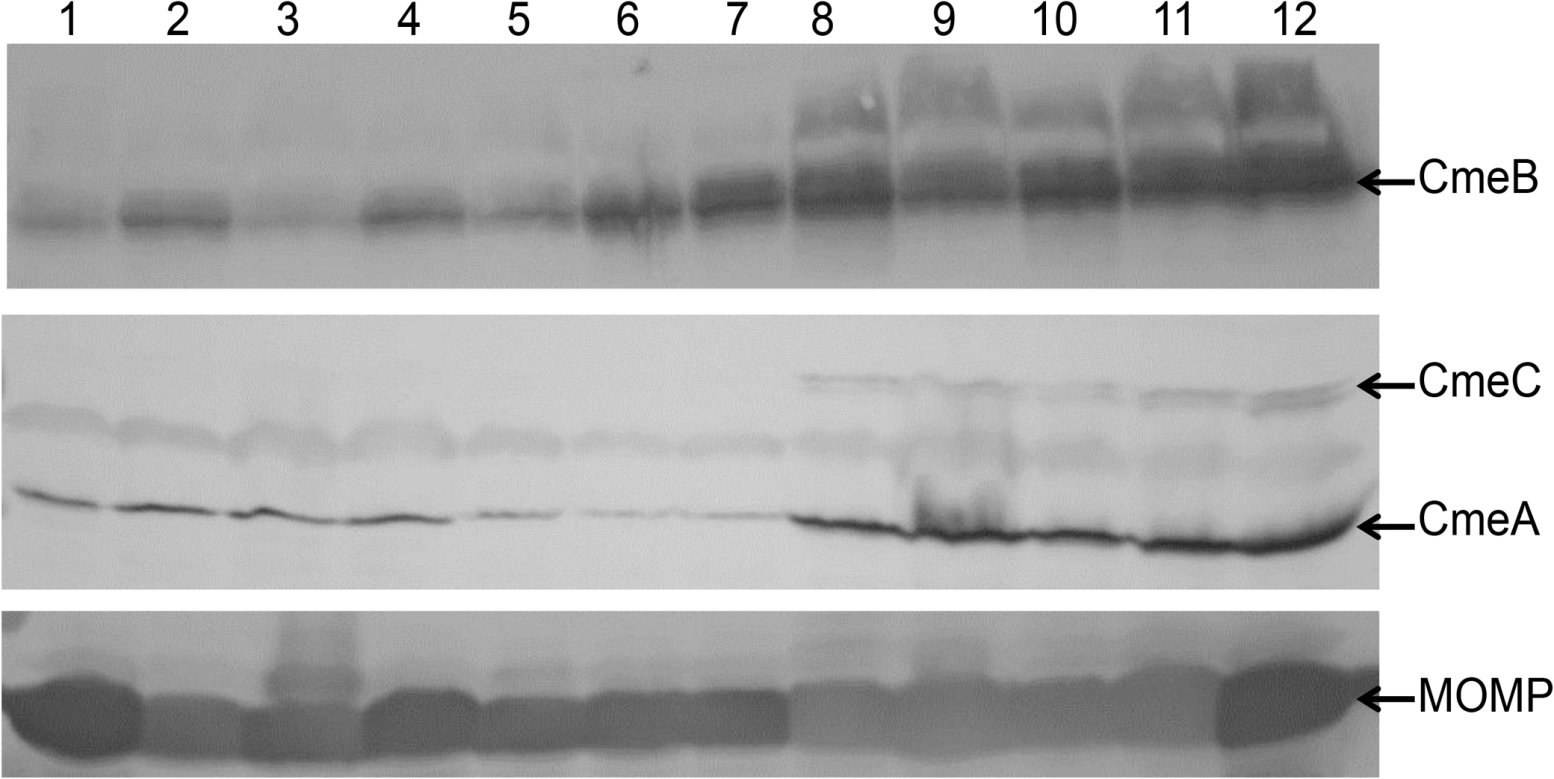
Differential expression of CmeABC in clinical *Campylobacter jejuni* isolates. Expression was determined by immunoblotting of whole cell proteins from NCTC 11168 (lane 1), clinical isolates (lanes 2–11), and 11168Δ*cmeR* (lane 12) with anti-CmeB, anti-CmeC, anti-CmeA, and anti-major outer membrane protein (MOMP) antibodies. These broiler isolates in lanes 2 to 11 are CB1:6, CB1:14, CB 1:18, CB2:6, CB2:8, CB2:11, CB3:1, CB3:5, CB 3:14, and CB3:21. Isolates CB2:8, CB2:11, CB 3:1, CB3:5, CB3:14 and CB3:21 (lanes 6–11) were designated as having overexpression levels of CmeABC and isolates CB1:6, CB1:14, CB 1:18, and CB2:6 (lanes 2–5) as having wild-type levels of CmeABC. The major outer membrane protein (MOMP) was used as an internal control.

Initial screening for phenotypic classification of CmeABC expression was done through immunoblotting (Fig 1) and real time RT-PCR for expression of *cmeB*. Analysis of the 64 *C*. *jejuni* isolates for *cmeB* expression identified 43 isolates with overexpression levels (OEL) of CmeABC and 21 isolates with wild-type expression levels (WEL) of CmeABC. The region spanning from *cmeR* to *cmeA* was sequenced for all OEL isolates, 4 of the WEL isolates, and *C*. *jejuni* strain 81–176 to identify genetic mutations that were potentially associated with the differential *cmeABC* expression (S1 Table). All isolates except 1 OEL isolate harbored mutations in the sequenced region compared to the same region in strain NCTC 11168.

**Fig 2:**
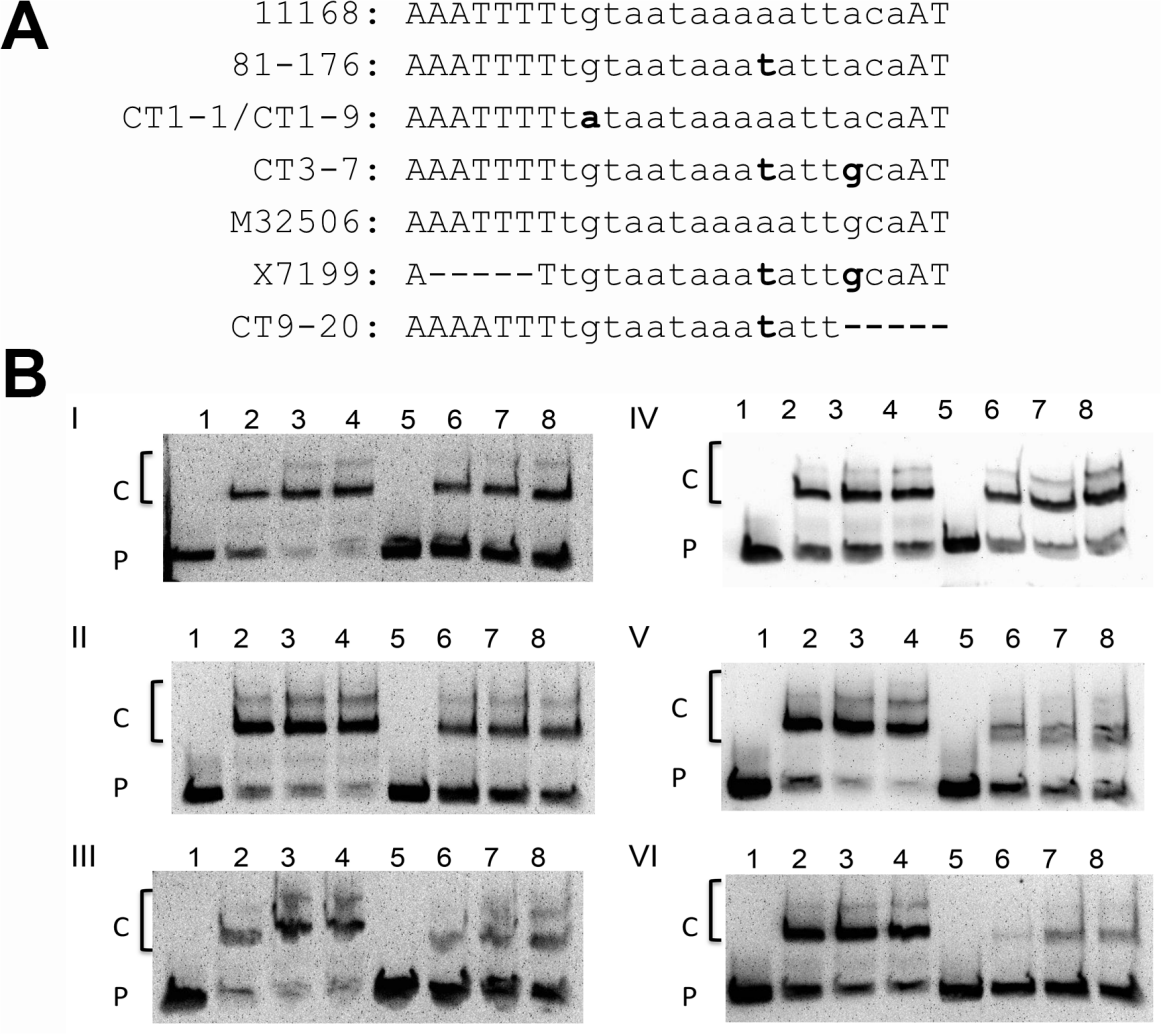
Binding of CmeR to variants of the cmeABC promoter in different isolates. (A) Sequence alignment of the cmeABC promoter region illustrating the 16-base inverted repeat of the CmeR binding site shown in lowercase letters. The strain names are listed on the left of each sequence. All mutations differing from the 11168 promoter are highlighted in bold. (-) indicates a deleted base. (B) EMSA showing the binding of rCmeRSS to different promoter variants. The control probes include the NCTC 11168 probe (lanes 1–4) in panels I-III and the 81–176 probe (lanes 1–4) in panels IV to VI. The variant promoter probes include CT1:1 (panel I, lanes 5–8), CT1:9 (panel II, lanes 5–8), M32506 (panel III, lanes 5–8), X7199 (panel IV, lanes 5–8), CT3:7 (panel V, lanes 5–8), and CT9:20 (panel VI, lanes 5–8). For each probe, the amount of rCmeRSS used for the each reaction was 0 (lanes 1 and 5), 60 (lanes 2 and 6), 120 (lanes 3 and 7), and 180 ng (lanes 4 and 8), respectively. The rCmeRSS-DNA complexes are indicated with a “C” and the unbound promoter probe is indicated with a “P”.

To refine the identification of mutations mediating differential *cmeABC* expression, the *cmeABC* promoter was analyzed for mutations unique to the OEL isolates and *cmeR* was analyzed for DNA polymorphisms resulting in amino acid changes. The CmeR binding site of the *cmeABC* promoter contains an A to T substitution at base 10 of the 16 base inverted repeat in strain 81–176 (Fig 2A) that is considered a natural variation (81–176 variation) [6]. Among the clinical isolates, 34 carried this mutation. Isolates that contained only the 81–176 variation in the CmeR binding site were excluded from further analysis. There were 14 OEL isolates carrying mutations within the CmeR binding site of the *cmeABC* promoter other than the 81–176 A to T substitution (Fig 2A). These isolates also carried amino acid mutations in CmeR. All of the observed amino acid mutations were also seen in isolates with no mutations in the *cmeABC* promoter. These 14 isolates were categorized as *cmeABC* promoter mutants.

Analysis of *cmeR* for DNA polymorphisms found numerous mutations leading to amino acid changes in CmeR. DNA polymorphisms that did not result in amino acid changes in CmeR were excluded. Three isolates, M63885, T37957A, and CT9:7 harbored unique mutations in CmeR (Table 3). Other OEL isolates carried amino acid changes in CmeR that were also seen in WEL isolates. Five OEL isolates and one WEL isolate with a unique combination of substitutions at residues 144, 183 and 207 were also selected for analysis (Table 3). None of these isolates, except X7199, had mutations in the *cmeABC* promoter. Together these 9 clinical isolates were categorized as *cmeR* mutants.

### Mutation of the CmeR binding site affects *cmeABC* expression in clinical isolates

Fig 2A illustrates the region of the *cmeABC* promoter containing the CmeR binding site. Two categories of substitutions were observed in the CmeR binding site: 5 isolates with a G to A substitution at base 2 of the inverted repeat and 7 isolates with an A to G substitution at base 14. The 81–176 variation (A to T at base 10) is found in 7 isolates carrying the substitution at base 14 and one isolate carrying the substitution at base 2. Two isolates carrying the 81–176 variation and a 5 base deletion in the *cmeABC* promoter were detected. The last 3 bases of the CmeR binding site and the following 2 bases are absent in isolate CT9:20 (Fig 2A). Isolate X7199 has a 5 base deletion 5’ to the CmeR binding site in addition to an A to G substitution at base 14 of the CmeR binding site (Fig 2A). These 14 isolates are considered *cmeABC* promoter mutants.

From the *cmeABC* promoter mutants, 6 representative sequences were selected for analysis by EMSA and transcriptional fusion (Fig 2A). The promoters from the isolates X7199 and CT9:20 were selected for their unique deletions. The M32506 and CT3:7 promoters both contain the A to G mutation at position 14, while the CT3:7 promoter also contains the 81–176 variation. The CT1:1 and CT1:9 promoters carry the G to A mutation at position 2.

The ability of CmeR to bind to the mutant *cmeABC* promoter sequences was assessed by EMSA. The selected mutant *cmeABC* promoter sequences from clinical isolates were paired with either the 11168 promoter (from strain NCTC 11168) (Fig 2B, panels I to III, lanes 1 to 4) or 81–176 promoter (Fig 2B, panels IV to VI, lanes 1 to 4) for use in EMSA based on the presence or absence of the A to T 81–176 variation in the CmeR binding site. Five of the 6 mutant promoters, CT1:1, CT1:9, M23506, CT3:7, and CT9:20 (Fig 2B, panels I, II, III, V, and VI respectively, lanes 5 to 8) showed decreased binding to rCmeRSS as manifested by the increased amounts of unbound probe and/or decreased amounts of probe- rCmeRSS complexes. Specifically, the CT1:1 (Fig 2B, panel I, lanes 5 to 8), CT1:9 (Fig 2B, panel II, lane 5 to 8) promoters showed increased amounts of free probe. The M32506 (Fig 2B, panel III, lane 5 to 8), CT3:7 (Fig 2B, panel V, lane 5 to 8), and CT9:20 (Fig 2B, panel VI, lane 5 to 8) probes display decreased intensity in the CmeR-DNA complexes and increased amounts of free probe. The CT9:20 *cmeABC* promoter (Fig 2B, panel VI, lanes 5–8) showed the largest reduction in CmeR binding (Fig 2B, panel VI lanes 7–8). The X7199 promoter displayed no difference in binding to rCmeRSS compared to the 81–176 promoter (Fig 2B, panel IV). These results suggest that most of the examined mutations in the *cmeABC* promoter sequence affected binding by CmeR.

**Fig 3:**
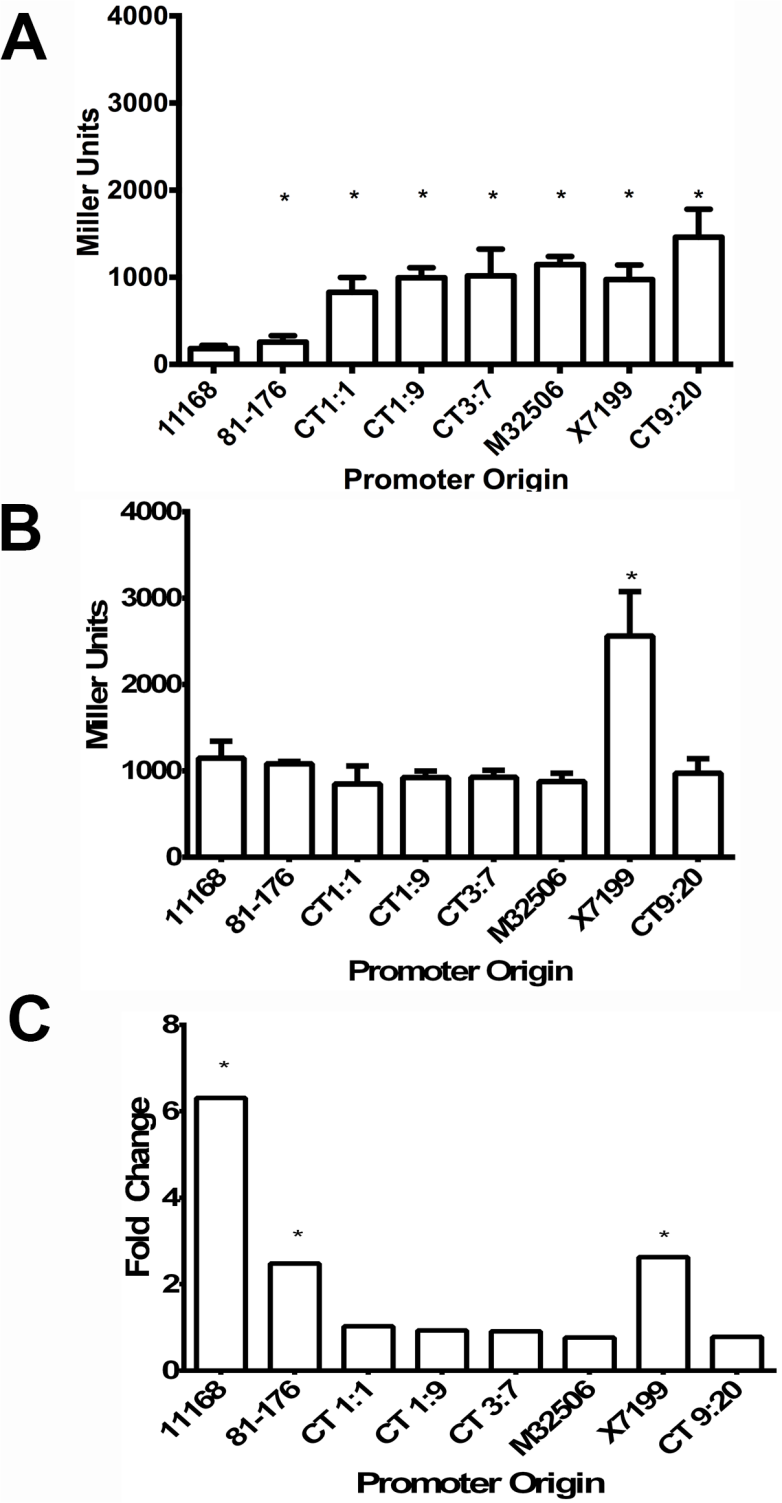
Effect of various mutations in CmeR and the promoter region on transcription of *cmeABC*. The names of the promoters used in the transcriptional fusions and β-galactosidase assays are indicated under each panel. Each promoter was assayed in the wild-type 81–176 background (A) and the 81–176ΔcmeR background (B). The data represent means with standard deviation from three independent experiments. The relative difference in transcription (fold change) due to repression by CmeR for each promoter is shown in (C) and was determined by comparison of transcription in the absence of CmeR (B) to the presence of CmeR (A). The unpaired Student’s t-test with Welch’s correction was used for comparison of the means with significance set at 0.05.

To further quantify the effect of the promoter mutations on *cmeABC* expression, transcriptional fusion of the mutant *cmeABC* promoters to the promoterless *lacZ* gene was performed in the presence and absence of CmeR. In the 81–176 wild-type background, CmeR is expressed and binds to the *cmeABC* promoter, repressing its transcription. The 81–176Δ*cmeR* strain is an isogenic mutant that does not express CmeR, resulting in a loss of repression for the *cmeABC* promoter. Without this repressor, *cmeABC* is overexpressed. Transcription from the 11168 promoter in the 81–176 wild-type background (CmeR is present) was defined as the basal level of *cmeABC* transcription and used as a control. In the presence of CmeR (81–176 wild-type background) (Fig 3A), all examined *cmeABC* promoters were transcribed. Compared with the base (11168 promoter), expression from the 81–176 promoter, CT1:1 promoter, CT1:9 promoter, CT3:7 promoter, M32506 promoter, X7199 promoter was 2.4-fold (*p* = 0.0096), 4.6-fold (*p* = 0.0232), 5.5-fold (*p* = 0.0073), 5.6-fold (*p* = 0.0425), 6.3-fold (*p* = 0.0036), and 5.4-fold *(p* = 0.0150) higher, respectively. These increases are indicative of decreased repression by CmeR due to the mutations in the *cmeABC* promoters. Notably, the CT9:20 promoter with the deletion of the last 3 bases of the CmeR binding site, had the highest increase in transcription at 8-fold (*p* = 0.0208) over the basal levels, consistent with its most obvious reduction in binding by CmeR on EMSA.

Transcription from the CT1:1 and CT1:9 promoters, which had the same sequence (Fig 2A) but was carried in different isolates, was not significantly different when compared to each other. This indicates that choosing a single representative isolate is sufficient to evaluate the other isolates in the group. The transcription from the X7199 and CT3:7 promoters, which had the same two point mutations in the CmeR-binding site (Fig 2A), was not significantly different between the two promoters, although there was an additional mutation (5-base pair deletion) upstream of the CmeR binding site in the X7199 promoter. These results suggest that the substitutions in the CmeR binding site alone (represented by CT3:7) is sufficient to alter expression of *cmeABC*.

In the absence of CmeR, transcription for the 11168 and 81–176 promoters were elevated, and the levels of transcription among the promoters except X7199 were not significantly different (*p* > 0.05) (Fig 3B). Comparisons for each promoter in the presence (WT) and absence (Δ*cmeR*) of CmeR showed that deletion of CmeR significantly increased expression (*p <*0.05) of the *cmeABC* promoters from 11186 and 81–176 (Fig 3C). Transcription in the absence of CmeR (81–176 Δ*cmeR* background) showed a 6.3-fold increase for the 11168 promoter (*p* = 0.014), a 2.5-fold increase for the 81–176 promoter (*p* = 0.0039), and a 2.6-fold increase for the X7199 promoter (*p* = 0.0367) (Fig 3C). The smaller change for the 81–176 and X7199 promoters compared to the 11168 promoter is likely due to already elevated expression in the wild-type background mediated by the CmeR binding site mutations (Fig 3A). Compared to their transcription in the *cmeR* wild-type background, the expression of CT1:1, CT1:9, CT3:7, M32506, and CT9:20 promoters were not significantly different (*p* > 0.05), suggesting that inactivation of *cmeR* did not further increase transcription from these promoters (Fig 3C).

### Varied expression levels of *cmeR* in clinical isolates

**Fig 4:**
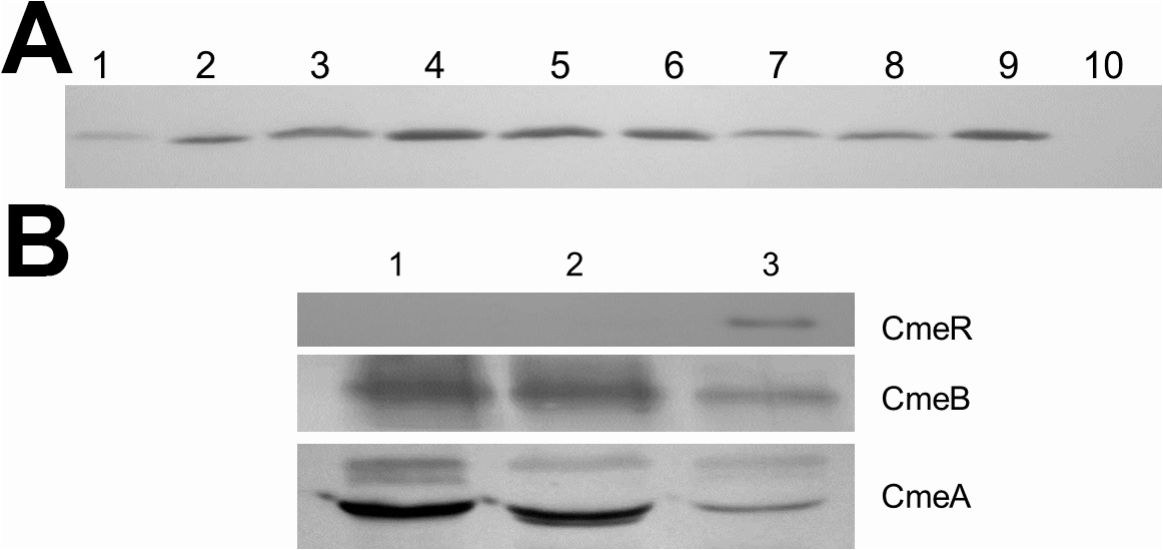
Expression of CmeR in various isolates and its correlation with CmeABC expression. (A) Immunoblotting of whole cell proteins from NCTC 11168 (lane 1), clinical isolates (lanes 2–9), and 11168Δ*cmeR* (lane 10) with the anti-CmeR antibody. The clinical isolates in lanes 2 to 9 are M63885, CT9:7, CB2:6, CB2:8, CB2:11, S13530, T37957A, and X7199, respectively. (B) Immunoblotting of whole cell proteins from 11168ΔcmeR (lane 1), CT2:2 (lane 2), and NCTC 11168 (lanes 3) with anti-CmeR, anti-CmeB, and anti-CmeA antibodies.

Several DNA polymorphisms were detected in the *cmeR* gene of the clinical isolates, resulting in amino acid changes in this regulatory protein (Table 3). Immunoblotting of whole cell proteins was performed to determine the CmeR expression level from 8 OEL isolates and 1 WEL isolate harboring representative mutations. The anti-CmeR antibody detected the CmeR protein from 8 of the 9 *cmeR* mutants (Fig 4A). The remaining *cmeR* mutant isolate (CT2:2) did not produce a band reactive with the antibody (Fig 4B, lane 2), suggesting that the CmeR protein was not translated in this isolate. CT2:2 contained a T insertion after base 583 in *cmeR*, resulting in a frame shift and premature truncation (Table 3). Additional immunoblotting failed to detect any portion of the truncated CmeR from CT 2:2.

To confirm the results of immunoblotting, *cmeR* expression was evaluated by real time RT-PCR. Real time expression levels of *cmeR* varied dramatically among the 9 isolates. Expression ranged from 0.006 to 33 fold of that in NCTC 11168 (Table 3), however this was not correlated with CmeR expression levels on immunoblotting (S2 Table). The transcript level of *cmeR* from CT2:2 were negligible at 0.006 fold of that in NCTC 11168, consistent with the lack of protein expression as detected by immunoblotting (Fig 4).

*cmeR* and *Cj0369c* form a two-gene operon and share a single promoter located in front of *Cj0369c* [13]. The predicted promoter for the *Cj0369c-cmeR* operon contains an inverted repeat with two half sites separated by a 12-base spacer that may represent an unknown regulatory mechanism [13]. Sequence analysis of this region was performed on several isolates to determine if mutations occurred and if they could be correlated with the varying *cmeR* expression levels identified by real time RT-PCR. Mutations of the *Cj0369c-cmeR* promoter were found in some isolates after comparison to the sequence of NCTC 11168 and were divided into 2 groups (S2 Table). The first group carried a single base deletion one base 5’ to the second half site of the inverted repeat in strains 81–176, T37957A, and E46972. The second group, consisting of isolates X7199, W52546, and S13530, contained a T insertion in the second half site of the inverted repeat after the eighth base and a G to A substitution at base 5 of the spacer. The *Cj0369c-cmeR* promoter from CT2:2 had no mutations, suggesting that transcription from this promoter is unlikely the source of the decreased production as observed by immunoblotting and real-time PCR.

Transcriptional fusion of representative *Cj0369c-cmeR* promoters from strains 81–176, X7199, and NCTC 11168 was performed to determine if the observed polymorphisms affected transcription. The 81–176, NCTC 11168, and X7199 promoters produced low levels of transcription (mean Miller units in the range of 8–15), which were not significantly different (p > 0.05) (S1 Fig), suggesting that these mutations were not associated with the expression levels of *cmeR*. However, this was not consistent with the real time *cmeR* expression data (Table 3). The mutations observed in the *Cj0369c-cmeR* promoter for X7199 and the S13530 were identical but, *cmeR* expression was 10.9 and 0.2 fold of NCTC 11168 respectively. The reason for this discrepancy is unclear.

### Truncation, but not amino acid substitution affected CmeR binding to the *cmeABC* promoter

**Fig 5:**
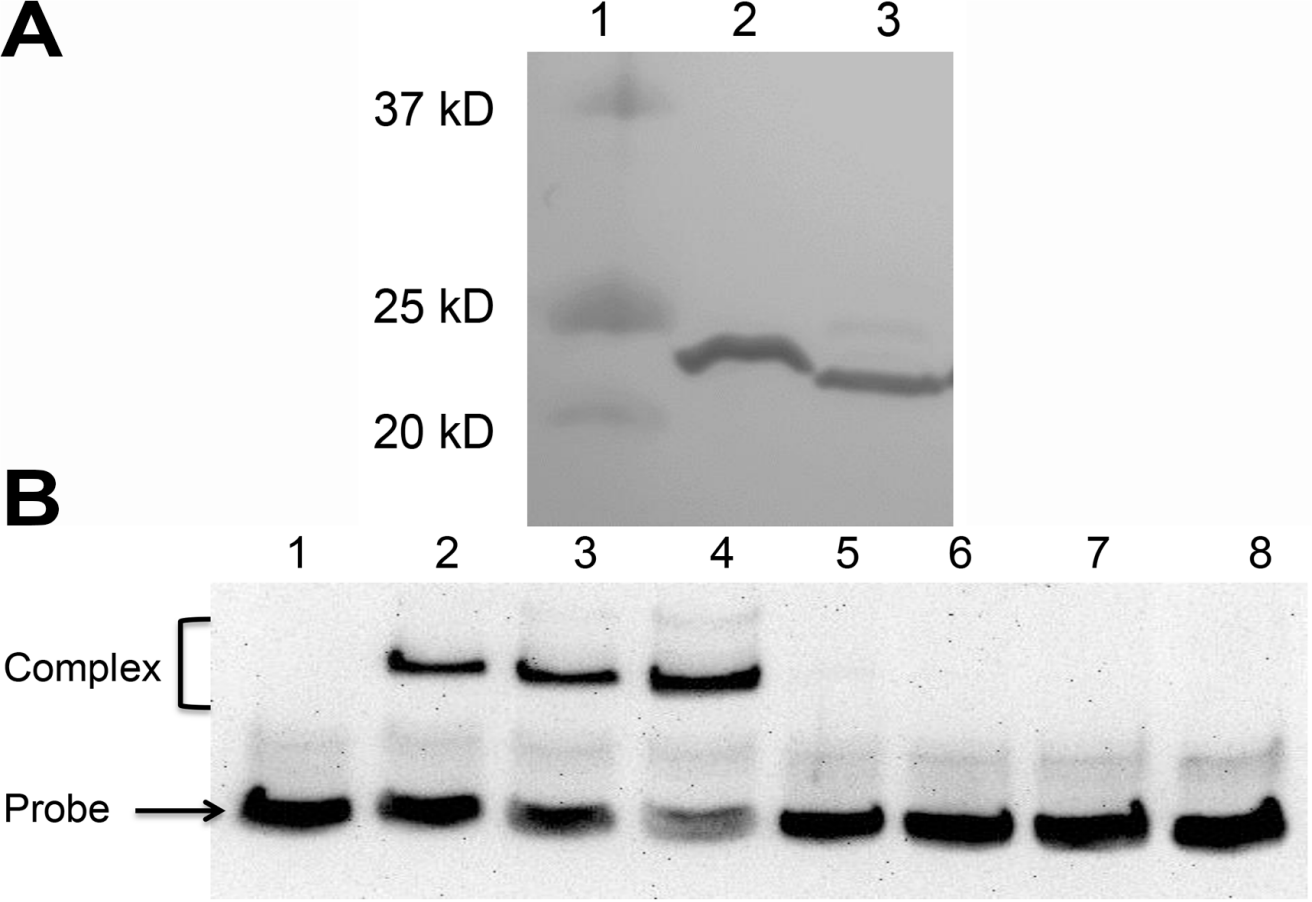
Inability of recombinant CmeR from isolate CT2:2 to bind to the promoter DNA of *cmeABC*. (A) Immunoblotting of purified rCmeRSS (lane 2; wild-type CmeR with C69 and C166 replaced with serine) and rCmeR-tr (lane 3; truncated CmeR after residue 193 from isolate CT2:2) with the anti-CmeR antibody. Lane 1 is the protein standard ladder. (B) EMSA showing binding of the 11168 cmeABC promoter by rCmeRSS (lanes 1–4) and rCmeR-tr (lanes 5–8). Proteins were added at 0, 60 (lanes 2 and 6), 120 (lanes 3 and 7), 180 ng (lanes 4 and 8). The locations of protein-DNA complexes and the probe are indicated.

The amino acid changes in CmeR observed in clinical isolates were categorized into 5 groups represented by the 9 *cmeR* mutant isolates (Table 3). Site-directed mutagenesis and recombinant CmeR production were performed for 3 representative isolates with amino acid substitutions and a single isolate with substitution and truncation of CmeR (Table 3). The 4 proteins produced were named rCmeR-tr, rCmeR-IK, rCmeR-K and rCmeR-R. The rCmeR-IK from M63885 contains 2 amino acid substitutions at residues 6 and 159 replacing threonine with isoleucine and glutamate with lysine, respectively. The rCmeR-K from T37957A and rCmeR-R from CT9:7 contain single amino acid substitutions of the glutamate at residue 84 for lysine in rCmeR-K and the proline 183 residue for arginine in rCmeR-R. The rCmeR-tr from isolate CT2:2 contains a glycine to alanine substitution at residue 144 and a nucleotide insertion after base 583 resulting in pre-mature truncation of CmeR to 193 amino acids (the full-length CmeR is 210 amino acids). The S207G substitution observed in CT2:2 occurs downstream of the truncation and was not expected to affect CmeR function. rCmeR-tr was detected by immunoblotting with the anti-CmeR antibody and presented as a band of 23 kD (Fig 5A, lane 3), slightly smaller than the full-length rCmeRSS at 24kD (Fig 5A, lane 2), consistent with the predicted truncation.

EMSA was performed with all 4 mutant proteins to assess the binding to the NCTC 11168 *cmeABC* promoter (11168 promoter). The rCmeR-R, rCmeR-IK, or rCmeR-K proteins bound to the *cmeABC* promoter in a manner similar to the rCmeRSS control (S2 Fig). However, rCmeR-tr failed to bind to the *cmeABC* promoter at all tested concentrations (Fig 5B, lanes 5–8) suggesting that the truncation abolished the ability to bind promoter DNA.

### Differences in antimicrobial susceptibility

*In vitro* antimicrobial susceptibilities did not differ between the OEL and WEL isolates in the presence or absence of bile for most of the tested antimicrobials except for chloramphenicol. Without bile, the median MIC for chloramphenicol was 4 μg/mL for both the OEL and WEL isolates (Table 4). However, the distribution of MICs around the median was significantly different between the two phenotypic groups (*p* < 0.05). The WEL isolates had chloramphenicol MICs of 2 to 4 μg/mL with 67% of the isolates at 4 μg/ml, while the OEL isolates had MICs ranging from 2 to 16 μg/mL with 21% of the isolates at 8 and 16 μg/mL (Table 4). With bile in the testing media, the MIC distribution between the two phenotypes was also significantly different (*p* <0.05) (Table 4). Although the median remains at 4 μg/mL for both groups, 81% of the OEL isolates were at this MIC, while 58% of the WEL isolate were at this MIC. Both OEL and WEL isolates have MIC ranges of 2 to 8 μg/mL after addition of bile. Compared to the non-bile media, addition of bile shifted the MIC to the upper end for WEL isolates and shifted the MIC to the median the for OEL isolates.

**Table 4:** Chloramphenicol MIC distribution (% for each MIC) among the tested isolates.

MIC	2 μg/mL	4 μg/mL	8 μg/mL	16 μg/mL
MH agar				
WEL	33	67	0	0
OEL	42	37	19	2
MH agar with ox-bile^1^				
WEL	33	58	8	0
OEL	16	81	2	0

^1^Ox bile 12,500 μg/mL

### Emergence of ciprofloxacin-resistant mutants

**Fig 6:**
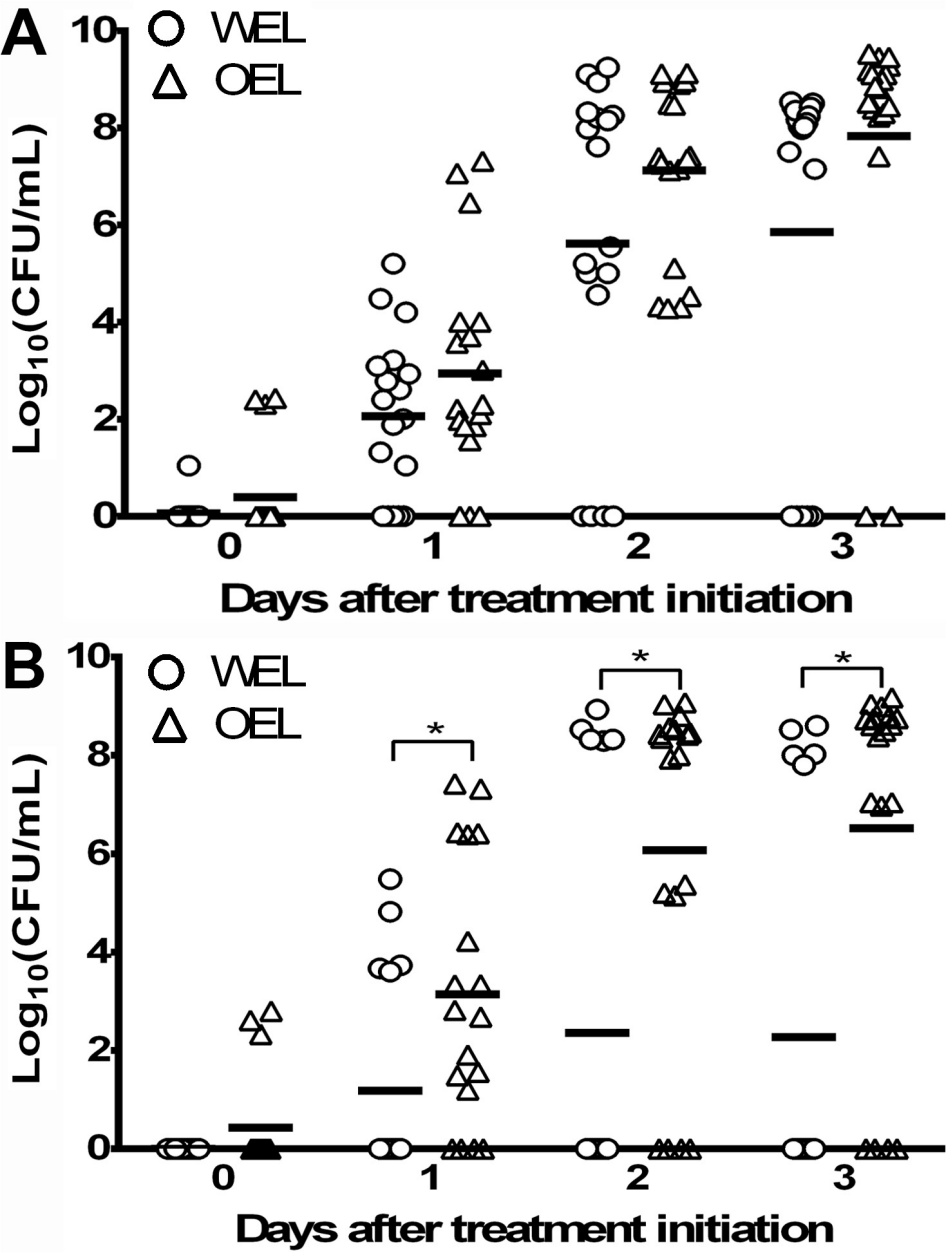
Emergence of ciprofloxacin-resistant mutants from WEL (circle) and OEL (triangle) isolates during treatment with ciprofloxacin. In panel A, the experiment was performed with an initial inoculum of 10^7^ CFU/ml of each isolate, while in panel B, the inoculum was 10^6^ CFU/mL for each isolate. The culture medium was MH broth containing 4 μg/mL of ciprofloxacin. Three WEL and OEL isolates were used in each experiment with cultures prepared in triplicate. Each point represents the number of ciprofloxacin-resistant mutants from a single culture. Bars represent mean log_10_ CFU/mL for each group. Means for each phenotypic group were compared for each day with multiple unpaired Student’ t-tests and Holm-Šídák method for multiple comparisons. The significance level was set at 0.05.

The spontaneous mutation rate to ciprofloxacin was examined for selected isolates using the fluctuation assay. Although there was a trend that the mutation rate was higher in OEL than in WEL, the difference was not statistically significant (*p*> 0.05) (S3 Fig). However, OEL isolates showed increased emergence of ciprofloxacin-resistant (Cip^R^) mutants during *in vitro* treatment (Fig 6). Two experiments were performed with the initial inoculum levels of 10^7^ and 10^6^ CFU/mL, respectively. For the inoculums at 10^7^ CFU/mL, there were no significant difference in the mean numbers of pre-existing Cip^R^ mutants between the WEL and OEL cultures on day 0 (Fig 6A). Cip^R^ populations from both WEL and OEL cultures expanded over days 1 to 3. The mean Cip^R^ mutant populations were 0.9 logs higher for OEL on day 1, 1.5 logs higher on day 2, and 2 logs higher than WEL on day 3. However, the means were not significantly different between the OEL and WEL groups (*p* > 0.05).

For the inoculum of 10^6^ CFU/mL, the means for pre-existing Cip^R^ mutants on day 0 were not significantly different for the WEL and OEL cultures. The mean Cip^R^ mutant numbers on day 1 were 2 logs (*p* = 0.0175) higher than the mutants in the WEL cultures. This trend continued on days 2 and 3 with OEL means being 3.7 logs (*p* = 0.0053) and 4.2 logs (*p* = 0.0016) higher than the WEL means, respectively. These results indicate OEL cultures produced significantly higher numbers of Cip^R^ mutants than the WEL cultures during ciprofloxacin treatment.

## Discussion

This study demonstrates that differential expression of CmeABC naturally occurs in *Campylobacter* isolates derived from different host species. The variable expression was linked to multiple mechanisms including mutations in the *cmeABC* promoter region and coding sequences of CmeR as well as decreased expression of CmeR, the repressor for the *cmeABC* operon. Additionally, there are unidentified mechanisms that also modulate *cmeABC* expression as some OEL isolates did not have any mutations in the known regulatory elements for *cmeABC*. Differential expression of *cmeABC* was associated with altered susceptibility to chloramphenicol and enhanced the emergence of Cip^R^ mutants under selection with ciprofloxacin. These findings suggest that differential expression of CmeABC may be selected under natural conditions and may facilitate *Campylobacter* adaptation to various environments.

Of the 64 isolates examined in this study, 43 (67%) were phenotypically classified as OEL isolates. CmeABC is normally repressed by CmeR, which binds to the promoter region of *cmeABC* [12]. Thus, mutations in CmeR and/or the *cmeABC* promoter sequence were investigated to determine the genetic basis associated with overexpression of *cmeABC* in these clinical isolates. Those mutations that occurred in the CmeR binding site or resulted in amino acid changes in CmeR and were absent from the majority of the WEL isolates were selected for detailed analysis. Additionally, those isolates harboring the same mutation as the one in the CmeR binding site of strain 81–176 were excluded from analysis as this mutation has been characterized previously [12]. Using these selection criteria, we selected 14 isolates harboring promoter mutations and 9 isolates harboring CmeR mutations for detailed analysis.

Most of the detected amino acid substitutions in CmeR did not affect its function as determined by EMSA. However, a single nucleotide insertion at the 3’end of the *cmeR* gene resulted in a frame-shift and presumably led to truncation of the CmeR protein in CT2:2. CmeR was not detected by immunoblotting in CT2:2 (Fig 4B) and the *cmeR* transcript level was also significantly reduced. Additionally, CT2:2 did not contain any mutations in the predicted *Cj0369c-cmeR* promoter region, excluding the possibility that lack of *cmeR* expression was due to altered transcription initiation. These findings suggest that the single nucleotide insertion could have destabilized the *cmeR* transcript and/or the frame shift rendered the CmeR protein unstable in C. *jejuni*, leading to the lack of CmeR in this isolate. However, a recombinant version of the truncated CmeR (rCmeR-tr) was successfully generated (Fig 5A), suggesting that this truncated CmeR is stable in the *E*. *coli* host. Interestingly, rCmeR-tr failed to bind to the promoter DNA of *cmeABC* as determined by EMSA (Fig 5B), suggesting that even if this truncated version is made in *C*. *jejuni*, it would not be able to control the expression of *cmeABC*. The lack of CmeR production and the inability of the truncated CmeR to bind to promoter DNA fully explain the overexpression of *cmeABC* in isolate CT2:2.

It is interesting that rCmeR-tr lost the ability to bind DNA despite the fact that the truncation occurred in the C-terminal end of CmeR and the DNA-binding domain remained intact. This could be explained using the known structural information of CmeR. CmeR functions as dimer *in vivo* and the crystal structure of CmeR identified that α helices 6, 8, 9, and 10 are involved in dimer formation [12, 15, 50]. The truncation in rCmeR-tr occurred in the α10 helix of CmeR. Thus, the truncation in rCmeR-tr likely affects dimer formation and ultimately the function of CmeR. This result suggests that the C-terminal sequence of CmeR is also important for its interaction with target DNA. Recently, *C*. *jejuni* ATCC 33560, a quality control strain used for antimicrobial susceptibility testing in *C*. *jejuni*, was found to contain a frame shift mutation in *cmeR*, which led to truncation of the CmeR protein [51]. The truncation occurs in α helix 8 of CmeR and presumably results in non-functional CmeR [51]. Together, these findings indicate that frame-shift mutations in CmeR occur under natural conditions. As CmeR is a pleiotropic regulator (regulating other genes in addition to *cmeABC*) [13], truncation of CmeR likely affects multiple functions in *C*. *jejuni*.

The majority of the mutations in the *cmeABC* promoter were found to affect *cmeABC* expression. The *cmeABC* promoter contains a 16 base inverted repeat that serves as the specific binding site for CmeR [12]. Mutations in the CmeR binding site within the *cmeAB*C promoter have been described previously after *in vitro* stepwise selection with erythromycin [14] and ciprofloxacin [12]. This study is the first to describe the occurrence of this type of mutation in *C*. *jejuni* isolates from natural sources and various hosts including humans, turkeys, and chickens. These mutations inhibited binding by CmeR, resulting in increased transcription from the *cmeABC* promoter. This was demonstrated by reduced binding of the mutant promoters by CmeR on EMSA (Fig 2B, panels I, II, III,V, and VI) and increased transcription of *cmeABC* as determined by transcriptional fusion assays (Fig 3). In addition to the substitution, deletions within the CmeR binding site were found in one isolate, CT9:20. This promoter showed the greatest inhibition of CmeR binding on EMSA and the largest increase in transcription in the presence of CmeR (Fig 3A). These findings indicate that mutations in the *cmeABC* promoter commonly occur and these mutations influence the expression of this multidrug efflux pump.

Multiple mutations were also identified in a single isolate. For example, isolate X7199 harbored a substitution in the CmeR binding site and a 5 base deletion upstream of the CmeR binding site (Fig 2A). This isolate also contained mutations in *cmeR* (Table 3). The *cmeABC* promoter in this isolate was identical to the promoter in CT3:7 except for a 5-base pair deletion upstream of the CmeR binding site (Fig 2A). While the CT3:7 promoter demonstrated reduced binding to CmeR on EMSA (Fig 2B, panel V), the X7199 promoter had similar binding as the 81–176 *cmeABC* promoter (Fig 2B, panel IV). However, both the X7199 and CT3:7 *cmeABC* promoters demonstrated similar, elevated expression by transcriptional fusion assay compared with the 11168 promoter in the presence of CmeR (Fig 3A). This discrepancy between the results of EMSA and transcriptional fusion suggests that the EMSA assay has a lower sensitivity than the transcriptional fusion assay, or alternatively, there is another regulatory mechanism that also modulates *cmeABC* expression. Indeed, when the X7199 promoter was examined in the absence of CmeR using transcriptional fusion (Fig 3B), its expression was significantly increased compared to its own expression level in the presence of CmeR (Fig 3C). This result is consistent with the EMSA result and suggests that the X7199 promoter is still under the repression by CmeR.

In some isolates, the OEL phenotype was not linked to the known mechanisms modulating *cmeABC* expression as there were no mutations detected in CmeR or the promoter region. Additionally, even though some isolates harbored mutations in CmeR, these mutations did not affect the function of CmeR. Furthermore, the regulation of the X7199 promoter cannot be fully explained by a CmeR-only-dependent mechanism. These findings suggest that there may be additional regulatory mechanisms modulating *cmeABC* expression. Previously, Lin *et*. *al*. 2005 also described a CmeR-independent mechanism modulating *cmeABC* expression [16]. Bile is a known inducer of CmeABC and mediates increased expression by altering the confirmation of CmeR, resulting in disassociation of CmeR from the *cmeABC* promoter [15, 16]. It was noticed that in the absence of CmeR, the expression of *cmeABC* was further induced by taurocholate, suggesting this bile compound induced expression of *cmeABC* through a CmeR- independent mechanism [16]. Taken together, observations from this study and previous reports suggest that multiple mechanisms modulate the expression of *cmeABC*.

The functional consequence of *cmeABC* overexpression was evaluated in relation to antimicrobial treatments. Antimicrobial susceptibility was unaffected by *cmeABC* overexpression for most of the tested antibiotics, with the exception of chloramphenicol. This was not surprising as a previous study using genetic manipulation revealed that overexpressing *cmeABC* by inactivating *cmeR* had a modest effect on MICs, but inactivation of *cmeABC* significantly increased the susceptibility of *C*. *jejuni* to antibiotics [6, 12]. For chloramphenicol, the OEL isolates displayed a larger range of MIC values than WEL isolates, with more MICs distributed above the median value (Table 4). This suggests that overexpression of CmeABC had an effect on the MIC distribution of chloramphenicol. Interestingly, when chloramphenicol MIC was measured in the presence of bile, the MICs of the WEL isolates shifted above the median value, while the MICs of the OEL isolates shifted toward the median value. This difference is probably due to the fact that bile is an inducer for *cmeABC* and the possibility that induction was more prominent in the WEL isolates than in the OEL isolates. For the OEL isolates, *cmeABC* was already overexpressed due to less inhibition by CmeR or other unidentified mechanisms. Thus, the bile-mediated induction through CmeR is expected to be less effective in the OEL isolates than in the WEL isolates.

Fluoroquinolones resistance in *Campylobacter* is mediated by DNA gyrase mutations and the function of CmeABC [6, 8, 9]. These two mechanisms function synergistically in mediating resistance to fluoroquinolones [9]. Additionally, CmeABC promotes the emergence of fluoroquinolone-resistant mutants under antibiotic selection [8, 23]. In this study, we examined the correlation between the OEL phenotype and ciprofloxacin resistance. It was found that the basal spontaneous mutation rate was not affected by overexpression of *cmeABC* as measured by the fluctuation assay. However, the OEL isolates showed higher level of emergence of CipR mutants under antibiotic selection (Fig 6). This was consistently shown by using two inoculation doses (106 and 107 CFU/mL). For the 107 CFU/mL inoculum, the difference between the OEL and WEL groups were obvious, but were not statistically significant. For the 106 CFU/mL inoculum, the numbers of CipR mutants from the OEL isolates were significantly higher than those from the WEL mutants. The lack of statistical significance with the 107 CFU/mL inoculum was probably due to the presence of pre-existing CipR mutants in the inoculum (Fig 6) that somewhat reduced the difference between the OEL and WEL groups. Thus, reducing the inoculum to 106 CFU/mL allowed clear detection of differences between the two groups. These results suggest that the OEL phenotype may facilitate *Campylobacter* to adapt to fluoroquinolone treatment by promoting the emergence of resistant mutants. This finding has practical implication as fluoroquinolone antibiotics are used for both human medicine and animal production. Thus, the detection of a large number of *C*. *jejuni* with an OEL phenotype from different host species might be the result of antibiotic usage that has served as a selection force for the OEL phenotype.

In summary, this study reveals that overexpression of CmeABC commonly occurs in *C*. *jejuni* isolates derived from various host species. The overexpression is mediated by multiple mechanisms including mutations in the *cmeABC* promoter sequence and in the CmeR coding sequence. Additionally, results from this study suggest that there are other unidentified mechanisms that modulate the expression of CmeABC. Overexpression of *cmeABC* promotes the development of resistant mutants upon treatment with fluoroquinolone antibiotics and may contribute to the survival and persistence of *C*. *jejuni* in animal reservoirs where antibiotics are commonly used. These findings provide new insights into the adaptive mechanisms of *C*. *jejuni* and further highlight the potential to control antibiotic resistant *Campylobacter* by targeting CmeABC.

## Supporting Information

S1 Fig Effect of various mutations in the promoter of *Cj0369c-cmeR* on the transcription of the operon. The promoters amplified from strains NCTC 11168, 81–176, and X7199 were fused to *lacZ* and were introduced into the wild-type 81–176 background. β-galactosidase assays were performed using the promoter fusions. The data represent means with standard deviation from three independent experiments. The data are not significantly different (*p>* 0.05).
(PDF)

S2 Fig Binding of recombinant CmeR to the promoter DNA of *cmeABC* as determined by EMSA. rCmeRSS (lanes 1–4 in all panels), rCmeR-IK (lanes 5–8 in A), rCmeR-K (lanes 5–8 in B), and rCmeR-R (lanes 5–8 in C) were used in the assay. Proteins were added at 0 (lanes 1 and 5), 60 (lanes 2 and 6), 120 (lanes 3 and 7), 180 ng (lanes 4 and 8). The locations of the protein-DNA complexes and the probe are indicated.
(PDF)

S3 Fig Spontaneous mutation rate of WEL and OEL isolates to ciprofloxacin (4μg/mL). Fluctuation assays were used to calculate the numbers of mutations per culture, m, (A) and the average mutation rate, μ, (B) by the Ma-Sandri-Sarkar Maximum Likelihood Estimator method. Each bar represents mean ± SEM for each phenotypic group. There is a trend that the mutation rate is higher in OEL than WEL, but the difference was not statistically significant (*p*> 0.05).
(PDF)

S1 Table GenBank accession numbers for sequences derived from the *C*. *jejuni* isolates. (PDF)

S2 Table Densitometric ratio of CmeR bands (Clinical isolates: NCTC 11186) for Fig 4A. (PDF)
