# Supplementary material for: Genetic Basis and Functional Consequences of Differential Expression of the CmeABC Efflux Pump in Campylobacter jejuni Isolates
Source: PLoS One. 2015 Jul 1;10(7):e0131534. doi: 10.1371/journal.pone.0131534 (PMC4488513; doi:10.1371/journal.pone.0131534)
Supplement: S1 Fig — The promoters amplified from strains NCTC 11168, 81–176, and X7199 were fused to lacZ and were introduced into the wild-type 81–176 background. β-galactosidase assays were performed using the promoter fusions. The data represent means with standard deviation from three independent experiments. The data are not significantly different (p> 0.05). (PDF) [file pone.0131534.s001.pdf]

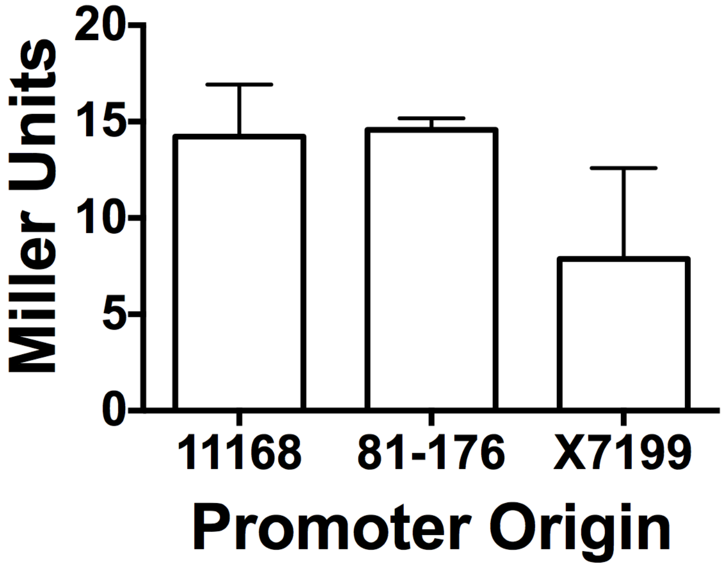

S1 Fig. Effect of various mutations in the promoter of *Cj0369c-cmeR* on the transcription of the operon. The promoters amplified from strains NCTC 11168, 81-176, and X7199 were fused to *lacZ* and were introduced into the wild-type 81-176 background. β -galactosidase assays were performed using the promoter fusions. The data represent means with standard deviation from three independent experiments. The data are not significantly different ($p > 0.05$).
