# Supplementary material for: Genetic Basis and Functional Consequences of Differential Expression of the CmeABC Efflux Pump in Campylobacter jejuni Isolates
Source: PLoS One. 2015 Jul 1;10(7):e0131534. doi: 10.1371/journal.pone.0131534 (PMC4488513; doi:10.1371/journal.pone.0131534)
Supplement: S2 Fig — rCmeRSS (lanes 1–4 in all panels), rCmeR-IK (lanes 5–8 in A), rCmeR-K (lanes 5–8 in B), and rCmeR-R (lanes 5–8 in C) were used in the assay. Proteins were added at 0 (lanes 1 and 5), 60 (lanes 2 and 6), 120 (lanes 3 and 7), 180 ng (lanes 4 and 8). The locations of the protein-DNA complexes and the probe are indicated. (PDF) [file pone.0131534.s002.pdf]

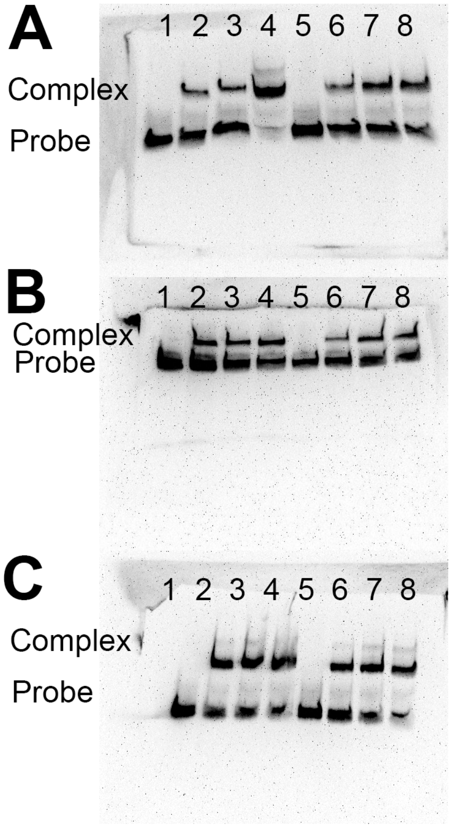

S2 Fig. Binding of recombinant CmeR to the promoter DNA of *cmeABC* as determined by EMSA. rCmeRSS (lanes 1-4 in all panels), rCmeR-IK (lanes 5-8 in A), rCmeR-K (lanes 5-8 in B), and rCmeR-R (lanes 5-8 in C) were used in the assay. Proteins were added at 0 (lanes 1 and 5), 60 (lanes 2 and 6), 120 (lanes 3 and 7), 180 ng (lanes 4 and 8). The locations of the protein-DNA complexes and the probe are indicated.
