# Supplementary material for: Genetic Basis and Functional Consequences of Differential Expression of the CmeABC Efflux Pump in Campylobacter jejuni Isolates
Source: PLoS One. 2015 Jul 1;10(7):e0131534. doi: 10.1371/journal.pone.0131534 (PMC4488513; doi:10.1371/journal.pone.0131534)
Supplement: S3 Fig — Fluctuation assays were used to calculate the numbers of mutations per culture, m, (A) and the average mutation rate, μ, (B) by the Ma-Sandri-Sarkar Maximum Likelihood Estimator method. Each bar represents mean ± SEM for each phenotypic group. There is a trend that the mutation rate is higher in OEL than WEL, but the difference was not statistically significant (p> 0.05). (PDF) [file pone.0131534.s003.pdf]

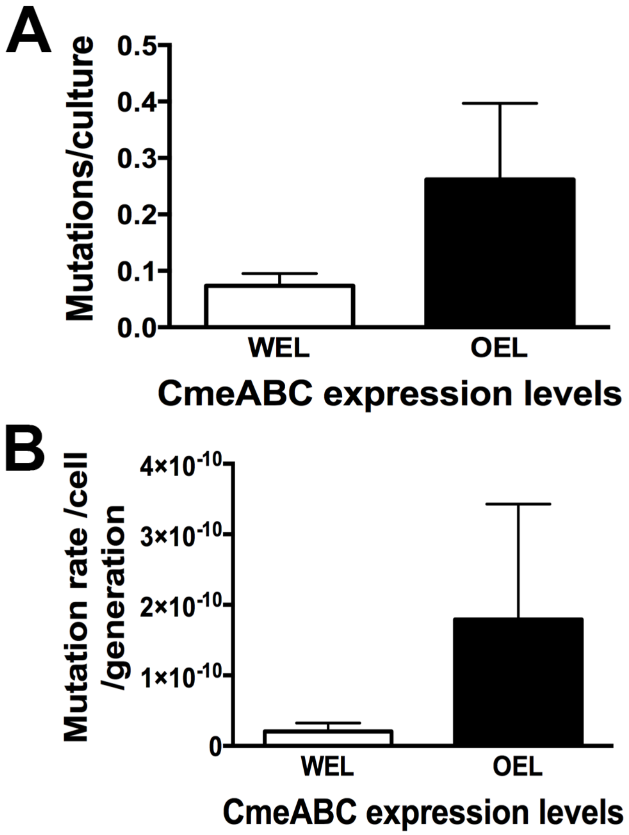

S3 Fig. Spontaneous mutation rate of WEL and OEL isolates to ciprofloxacin (4µg/mL). Fluctuation assays were used to calculate the numbers of mutations per culture, m, (A) and the average mutation rate, μ, (B) by the Ma-Sandri-Sarkar Maximum Likelihood Estimator method. Each bar represents mean ± SEM for each phenotypic group. There is a trend that the mutation rate is higher in OEL than WEL, but the difference was not statistically significant ($p > 0.05$).
