# Supplementary material for: Genetic Basis and Functional Consequences of Differential Expression of the CmeABC Efflux Pump in Campylobacter jejuni Isolates
Source: PLoS One. 2015 Jul 1;10(7):e0131534. doi: 10.1371/journal.pone.0131534 (PMC4488513; doi:10.1371/journal.pone.0131534)
Supplement: S1 Table — (PDF) [file pone.0131534.s004.pdf]

S1 Table. GenBank accession numbers for sequences derived from the *C. jejuni* isolates.

Region Sequenced	Strain	Accession Number
From *cmeR* to *cmeA*		
	CT1:1	KP856893
	CT1:9	KP856894
	CT2:2	KP856917
	CT3:7	KP856895
	CT6:8	KP856896
	CT6:16	KP856897
	CT6:18	KP856904
	CT7:2	KP856898
	CT7:20	KP856899
	CT9:7	KP856900
	CT9:14	KP856901
	CT9:20	KP856902
	CT9:21	KP856903
	CB1:14	KP856912
	CB2:6	KP856905
	CB2:8	KP856906
	CB2:11	KP856907
	CB3:1	KP856908
	CB3:5	KP856909
	CB3:14	KP856910
	CB3:21	KP856911
	CB4:11	KP856915
	CB4:22	KP856914
	CB8:14	KP856916
	CB8:15	KP856913
	M63885RIGA	KP856918
	M32506RIGA	KP856919
	M33323RIGA	KP856920
	M402RIGA	KP856921
	M76297RIGA	KP856922
	W11805RIGA	KP856923
	X39768RIGA	KP856924
	W64861RIGA	KP856925
	M37523RIGA	KP856926
	E46972RIGA	KP856927
	H49024RIGA	KP856928
	M36292RIGA	KP856929
	S13530RIGA	KP856930
	S47645RIGA	KP856931
	X7199RIGA	KP856932

	H30769RIGA	KP856933
	W52546RIGA	KP856934
	X77136RIGA	KP856935
	H2958RIGA	KP856936
	T37597ARIGA	KP856937
	T59822RIGA	KP856938
	W28752RIGA	KP856940
	81-176	KP856939
From *Cj0369c-cmeR* promoter		
	E46972	KP856888
	T37957A	KP856889
	X7199	KP856890
	W52546	KP856891
	S13530	KP856892
