# Supplementary material for: Genetic Basis and Functional Consequences of Differential Expression of the CmeABC Efflux Pump in Campylobacter jejuni Isolates
Source: PLoS One. 2015 Jul 1;10(7):e0131534. doi: 10.1371/journal.pone.0131534 (PMC4488513; doi:10.1371/journal.pone.0131534)
Supplement: S2 Table — (PDF) [file pone.0131534.s005.pdf]

S2 Table. Densitometric ratio of CmeR bands (Clinical isolates: NCTC 11186) for Fig. 4A.

Lane	Strain	CmeR ratio
1	NCTC 11168	1.000
2	M63885	3.466
3	CT9:7	4.680
4	CB2:6	7.590
5	CB2:8	6.641
6	CB2:11	5.114
7	S13530	2.617
8	T37957A	3.977
9	X7199	6.770
10	11168Δ*cmeR*	ND

ND- Not determined
